# SARS-CoV-2 RNA-dependent RNA polymerase as a target for high-throughput drug screening

**DOI:** 10.2217/fvl-2021-0335

**Published:** 2023-02-03

**Authors:** Jiahui Wu, Zhiqiang Chen, Xue Han, Qiaoqiao Chen, Yintao Wang, Tingting Feng

**Affiliations:** ^1^Institute of Biology & Medical Sciences, Jiangsu Key Laboratory of Infection & Immunity, Soochow University, Suzhou, 215123, Jiangsu Province, China; ^2^Department of Nuclear Medicine, The First Affiliated Hospital of Soochow University, Suzhou, 215000, Jiangsu Province, China; ^3^Department of Clinical Laboratory, the Affiliated Hospital of Qingdao University, 59 Haier Road, Qingdao, 266000, Shandong Province, China

**Keywords:** COVID-19, high-throughput screening, nsp12–nsp8–nsp7, RNA-dependent RNA polymerase, SARS-CoV-2

## Abstract

The ongoing COVID-19 pandemic caused by the SARS-CoV-2 has necessitated rapid development of drug screening tools. RNA-dependent RNA polymerase (RdRp) is a promising target due to its essential functions in replication and transcription of viral genome. To date, through minimal RNA synthesizing machinery established from cryo-electron microscopy structural data, there has been development of high-throughput screening assays for directly screening inhibitors that target the SARS-CoV-2 RdRp. Here, we analyze and present verified techniques that could be used to discover potential anti-RdRp agents or repurposing of approved drugs to target the SARS-CoV-2 RdRp. In addition, we highlight the characteristics and application value of cell-free or cell-based assays in drug discovery.

The COVID-19, caused by the SARS-CoV-2, poses a tremendous threat to global public health [1,2]. According to the WHO report, by 17 June 2022, there were more than 535 million confirmed cases with over 6.3 million deaths in more than 220 countries, areas or territories [3]. The SARS-CoV-2, coupled with SARS-CoV that emerged in 2003 and Middle East respiratory syndrome coronavirus in 2012, belong to the genus *Betacoronavirus* in the family of Coronaviridae of the order Nidovirales [4–6]. The genome of SARS-CoV-2 comprises of a single-stranded positive-sense RNA of approximately 30 kb containing a 5′ cap structure and a 3′ poly (A) tail, and has at least 14 open reading frames (ORFs) [6,7]. The viral replicase proteins are encoded by 5′-most ORF1a/1b, which accounts for about two-thirds of the entire genome length [8,9]. ORF1a and ORF1b can be translated into two huge polyproteins referred to as pp1a and pp1ab, occasioned by a -1 programmed ribosomal frameshift [8,9]. These polyproteins are cleaved into 16 non-structural proteins (nsps) by viral encoded main protease (M^pro^) or 3C-like proteinase (3CL^pro^) and one or two papain-like proteases (PL^pro^) [8–10]. Out of the 16 nsps, nsp1–nsp11 are derived from pp1a while nsp1–nsp10 and nsp12–nsp16 are a product of pp1ab. In addition, ORFs 2–14 encode four prominent structural proteins: spike (S), envelope (E), membrane (M) and nucleocapsid (N) proteins with nine putative accessory factors which account for the rest of the genome [8,9]. The invariant organization of the CoV's genome is 5′-replicase-S-E-M-N-3′ [9].

Among the viral enzymes, RNA-dependent RNA polymerase (RdRp), also referred to as nsp12, exhibits considerable potential as a promising target for antiviral drugs. The core structural features such as aspartates D760 and D761 in the active regions involved in nucleotide catalysis of the RdRp are highly conserved, paving the way for drug repurposing [11–13]. And the SARS-CoV-2 RdRp shares a striking 96% sequence homology with that of SARS-CoV, although the identity of their genome is about 80% [6,14]. Furthermore, there are no RdRp homologs in human cells, thus minimizing the risk of off-target effects or toxicity [15]. Moreover, RdRp plays an essential role in the life cycle of RNA viruses. And continuously emerging variants of SARS-CoV-2 boost the development of broad spectrum antiviral drugs [16–18]. Therefore, the RdRp is a valuable druggable target for CoVs and a variety of methods have been emerged and performed to identify RdRp inhibitors from the compound libraries, especially from approved antiviral drugs to speed the drug discovery process. However, there is a lack of updated summaries and analyses of these methods. Here, we review the structure and function of SARS-CoV-2 RdRp, and highlight advances in cell-free and cell-based assays in screening for potential inhibitors targeting the SARS-CoV-2 RdRp.

## The functions of SARS-CoV-2 RdRp

Upon entering the host cell, the replicase genes are translated into the nsps from the positive-sense gRNA that functions as mRNA. The replicase proteins are assembled into a multi subunit replication and transcription complex, whose primary constituent is the RdRp in double-membrane vesicles, the principal hubs for CoVs RNA synthesis [19,20]. Thereafter, the replication and transcription complex utilizes negative-strand intermediates of the genome to generate both progeny gRNAs and a set of nested sgRNAs [20]. It is important to note that the production of sgRNAs that serve as mRNAs for structural and accessory proteins is a unique discontinuous step, and is a hallmark of Nidovirales [21,22]. Eventually, S, E, M and N proteins along with the generated gRNAs form mature virions [22]. Thus, from the viral phases, it is feasible to conclude that the RdRp might be indispensable in the replication and transcription of the virus genome. The RdRp synthesizes the negative-strand RNAs, gRNAs and sgRNAs, essential components in the production of mature virions.

The RdRp has a polymerase domain which catalyzes the formation of phosphodiester bonds between ribonucleotides, and harbors a nidovirus N-terminal RdRp-associated nucleotidyltransferase (NiRAN) domain [11,23]. A previous structure-based study showed that ADP-Mg^2+^ occupies the NiRAN domain active site [24]. Further, Yan *et al.* showed that the NiRAN domain catalyzes the transfer of a GMP to the 5′-diphosphate end, forming the cap central structure, which accounts for its guanylyltransferase activity [25]. Moreover, nsp12 has been indicated to attenuate activation of type I IFN by inhibiting IRF3 nuclear translocation and this suppression is independent of its polymerase activity [26]. However, another study produced contradictory conclusions suggesting different experiment systems could mislead the results of the firefly luciferase reporter assay [27–29].

## The RdRp is dependent on nsp7 & nsp8 in RNA synthesis

Previous data from *in vitro* assays has shown that recombinant SARS-CoV nsp12 fused with a glutathione S-transferase tag displays weak and nonprocessive polymerase activity [30,31], contrary to the demand for synthesis of a large RNA genome *in vivo*, a phenomenon that might hinder development of antiviral drugs which target the RdRp. Subissi *et al.* observed that the processivity of nsp12-mediated RNA synthesis, defined as the number of nucleotides polymerized during a single encounter of the RNA polymerase with its template, is enhanced through formation of a complex where nsp7 and nsp8 act as cofactors [32]. Thus, the nsp12–nsp8–nsp7 complex forms the basic RNA synthesizing machinery, while other viral nsps such as nsp13, nsp14 and nsp16 remain irreplaceable for the RNA replication and transcription [13,33]. Using biochemical assays and reverse genetics system, the study evaluated the functions of several and critical nsp7 and nsp8 residues and showed that mutations on nsp7 amino acids (K7, H36 and N37) delay virus growth. Furthermore, substitution of three nsp8 residues, P183, R190 and K58, is toxic to the virus survival [32]. The first two residues interact with nsp12, while the last mediates the interaction between the polymerase complex and RNA. Besides, deletion of any of the regions encoding nsp7 or nsp8 lead to a lethal phenotype in murine hepatitis virus [34].

**Figure 1.**
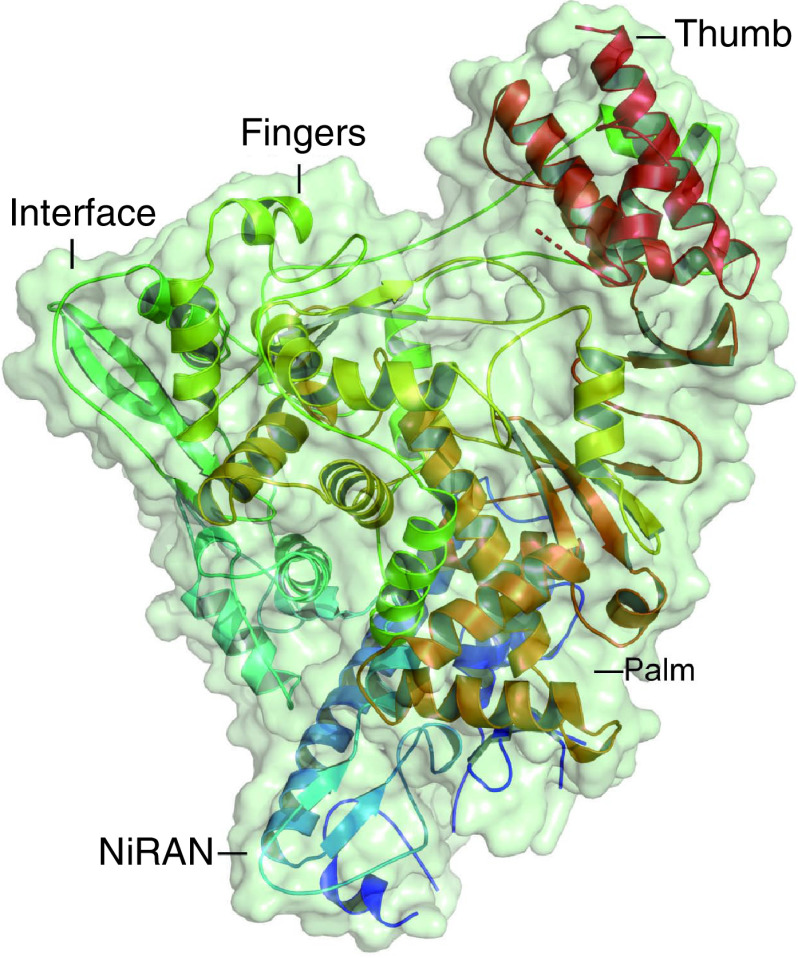
Structure of SARS-CoV-2 nsp12. The nsp12 subunit consists of a nidovirus N-terminal RNA-dependent RNA polymerase associated NiRAN domain, an interface domain, and a C-terminal polymerase domain composed of the fingers, palm and thumb subdomains. Protein Data taken from [37]. NiRAN: Nucleotidyltransferase.

Recent studies on high-resolution cryo-electron microscopy (cryo-EM) have revealed the structure of SARS-CoV-2 nsp12–nsp8–nsp7 complex, demonstrating the inextricable association between nsp12 and its cofactors [35–39]. Similar to the counterpart of SARS-CoV, SARS-CoV-2 nsp12 polymerase binds to a nsp7–nsp8 heterodimer and a second subunit of nsp8 with distinct binding sites [36,40]. As previously mentioned, the nsp12 subunit contains an N-terminal NiRAN domain and a C-terminal polymerase domain, bridged by an interface domain. The polymerase domain adopts a cupped ‘right hand’ configuration composed of the fingers (residues L366 to A581 and K621 to G679), palm (residues T582 to P620 and T680 to Q815) and thumb subdomains (residues H816 to E920) (Figure 1) [35,40]. The nsp7–nsp8 heterodimer binds to the thumb domain toward the NTP entry channel, while the second subunit of the nsp8 binds the interface domain close to the fingers domain and the RNA template binding channel [36]. Based on the conserved features of the nsp12, a newly N-terminal β hairpin domain has been identified. The domain inserts into the groove clamped by the NiRAN domain and the palm subdomain in the polymerase domain, resulting in tight junctions that stabilizes the overall architecture [35]. A study by Hillen *et al.* interrogated the interactions between the nsp12-8-7 complex and RNA based on their previous findings that there are over two turns of duplex RNA that engage the tripartite complex [38]. They showed a protruding RNA and long helical extensions in the nsp8 which might be sliding poles. The extensions slide along exiting RNA to block untimely dissociation of the nsp12, thus accounting for the functions played by the nsp8 in conferring processivity to the nsp12 during replication [38]. So far, there have been more than twenty structural data on the SARS-CoV-2 RdRp and associated factors [13].

## Assays used to screen for inhibitors of SARS-CoV-2 RdRp

The establishment of the SARS-CoV-2 nsp12–nsp8–nsp7 complex has facilitated drug discovery in cell-free and cell-based systems. Table 1 summarizes current assays used to screen for potential inhibitors of the SARS-CoV-2/SARS-CoV RdRp.

**Table 1.** Current assays for screening inhibitors of SARS-CoV-2/SARS-CoV RNA-dependent RNA polymerase.

Assay	Type	Target	Analysis	Screening result		Ref.
				Compound	Description	
Primer extension assay	Cell-free	SARS-CoV-2 RdRp	By urea-PAGE using a primer with a fluorescent label	Remdesivir-TP; 6-chloropurine-TP; 6-thio-GTP; 2′-C-methyl-GTP; 2′-amino-UTP; 2′-azido-UTP; ara-UTP	Can be incorporated into RNA very efficiently by RdRp	[41]
			By MALDI-TOF-MS using a primer without a label	3′-OMe-UTP; Carbovir-TP; Ganciclovir-TP; Stavudine-TP; Entecavir-TP; Biotin-16-dUTP; Cidofovir-DP; 2′-OMe-UTP; 2′-F, Me-UTP; 3′-F-dTTP; Tenofovir-DP	Terminate the RNA synthesis with varying efficiency	[42,43]
Fluorescence-based assay	Cell-free	SARS-CoV RdRp	By detecting the fluorescence signal	Anthracyclines; tetracyclines	IC_50_: from 0.34 to 44.5 μM; IC_50_: from 3.2 to 77.1 μM (compare with 3′dUTP IC_50_ (6.7 μM))	[44]
		SARS-CoV-2 RdRp		C646; BH3I1	IC_50_: 14.31 μM; IC_50_: 56.09 μM	[45,46]
Strand displacement assay	cell-free	SARS-CoV-2 RdRp	By detecting the fluorescence signal	C646, BH3I-1, GSK-650394 suramin	IC_50_: from 0.43 to 31 μM	[47]
Cell-based reporter assay	cell-based	SARS-CoV-2 RdRp	By measuring the luciferase activity	Quinoline and quinazoline derivatives; corilagin (RAI-S-37); 2-((indol-3-yl)thio)-N-benzyl-acetamides	Effectively inhibit the polymerase activity and resist the proofreading activity	[48–50]

DP: Diphosphate; RdRp: RNA-dependent RNA polymerase; TP: Triphosphate; Urea-PAGE: Denaturing polyacrylamide gel electrophoresis.

### Primer extension assay

**Figure 2.**
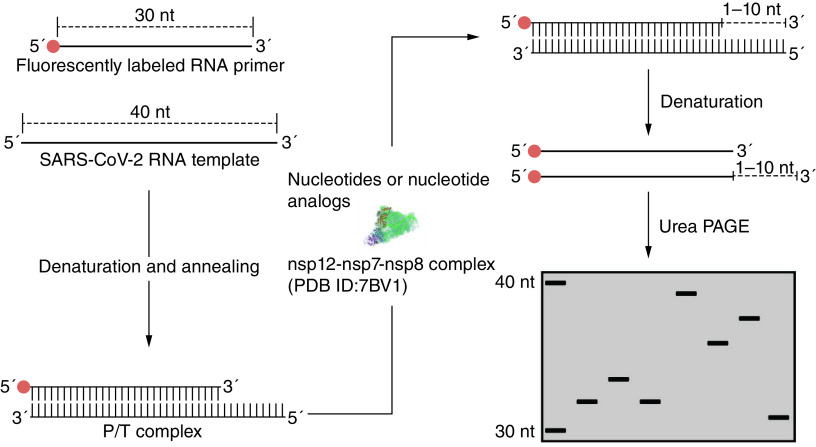
Primer extension assay. A fluorescently labeled RNA primer (30 nt) is annealed to an RNA template (40 nt) corresponding to the sequence of the 3′ end of the SARS-CoV-2 RNA genome to form a P/T complex. In the presence of nsp12–nsp7–nsp8 complex, the reaction is conducted with nucleotides or nucleotide analogs as substrates. After denaturation, the products are separated on a urea-PAGE gel. P/T: Primer-template; Urea-PAGE: Denaturing polyacrylamide gel electrophoresis.

Nucleoside analog inhibitors (NIs) have been in clinical treatment for over 50 years, and have become the mainstay of antiviral treatment options [51]. Since the NIs are prodrugs, they undergo cleavage in the liver via hepatic enzymes and are chemically transformed into a triphosphate form that directly acts on highly conserved and active RdRp sites [52]. Eventually, the NIs get incorporated into nascent RNA causing a lethal mutation or termination of chain elongation [53]. Currently, remdesivir (GS-5734), favipiravir, molnupiravir, ribavirin and penciclovir are promising NIs against RdRp for COVID-19 [53–56]. Hence, different studies have quantified and assessed the efficiency of incorporation of the NIs by SARS-CoV-2 RdRp. For instance, Lu *et al.* developed a simplified and robust *in vitro* nonradioactive primer extension assay to screen for NIs against RdRp (Figure 2) [41]. Briefly, the assay employs an RNA template corresponding to the 3′ end of the SARS-CoV-2 RNA genome sequence and a complementary RNA primer containing a 5′ end fluorescent label to construct a primer-template (P/T) duplex [41]. To perform the primer extension reaction, the annealed P/T complex is incubated with pre-assembled nsp12–nsp8–nsp7 complex in the reaction buffer, and the reaction is initiated by addition of natural ribonucleotide triphosphates as well as tested NIs to perform the incorporation of the NIs and chain-termination assays, respectively [41]. The P/T duplex extension products are then analyzed under denaturing polyacrylamide gel electrophoresis (urea-PAGE) following termination by the quenching solution [57]. Discrimination values, defined as relative efficiency of incorporation of the NIs versus natural nucleotides by the SARS-CoV-2 RdRp, are determined by estimation of the *K*_1/2_ values (the concentrations of substrates causing 50% primer extension), and are used to compare the chain-termination ability of a range of NIs [41,57]. The data showed higher incorporation efficiency of remdesivir triphosphate (RDV-TP) compared with that of ATP, and quadruple addition of RDV-TP gave rise to a robust termination [41]. To date, several primer extension assays using SARS-CoV-2 RdRp have been performed to examine the antiviral activity of NIs [42,43]. For instance, triphosphates of carbovir, entecavir, ganciclovir and stavudine acted as immediate chain terminators in the polymerase extension reaction [42]. Furthermore, this method was previously used to evaluate the antiviral ability of the NIs in targeting Zika and dengue virus RdRp and was applied to measure potential mitochondrial toxicity of NIs by human mitochondrial DNA-dependent RNA polymerase (POLRMT) [57–59]. In summary, primer extension assay has been a valuable method in screening nucleotide-based inhibitors of RdRp.

### Fluorescence-based assay

**Figure 3.**
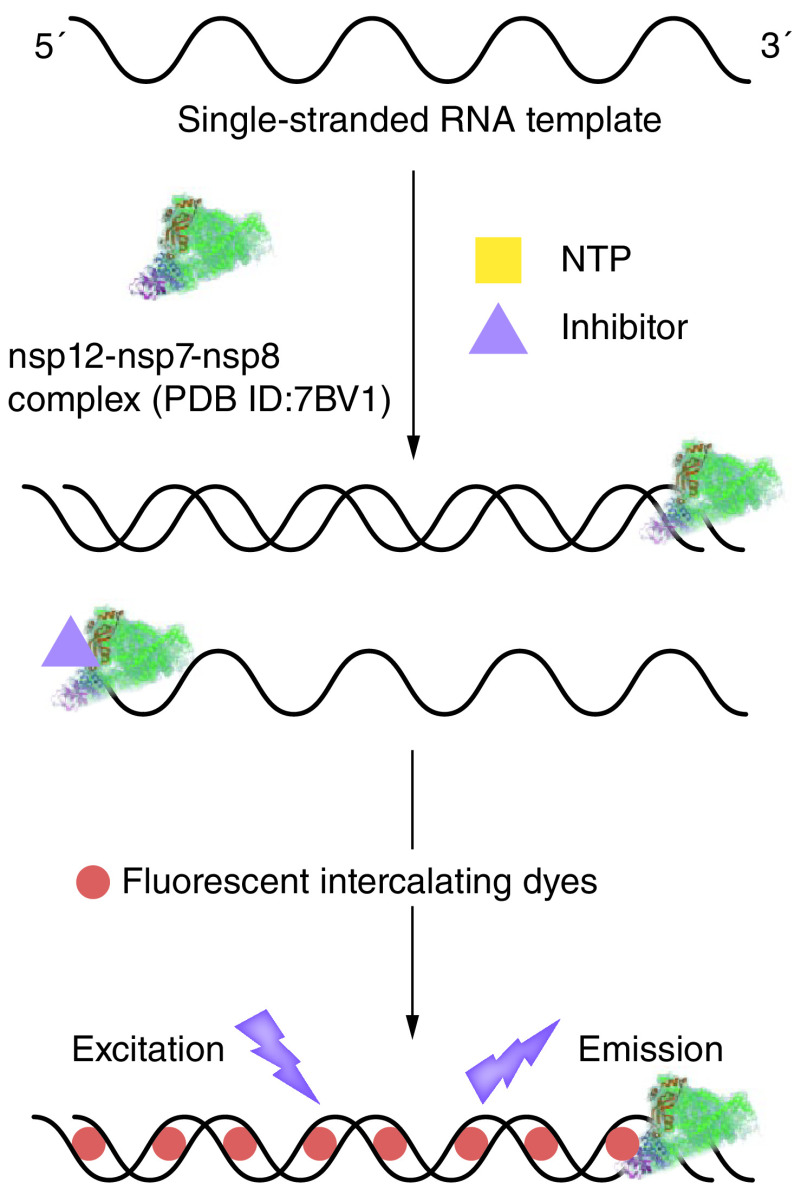
Fluorescence-based assay. With NTPs and putative inhibitors, a ssRNA template is incubated with nsp12–nsp7–nsp8 complex and then polymerase activity is analyzed by fluorescent intercalating dyes which can bind and quantify dsRNA. NTP: Nucleotide tri-phosphate; PDB ID: Protein Data Bank ID.

To accelerate the discovery of specific SARS-CoV-2 RdRp inhibitors, there is huge potential in the use of a reliable and cost-effective high-throughput screening (HTS) assay for antiviral compounds. To evaluate inhibitors of SARS-CoV RNA synthesis, a fluorescence-based HTS assay has been validated through screening of a small chemical library (1520 compounds) of US FDA approved drugs [44]. In this assay, a homopolymeric adenine RNA template and the SARS-CoV RNA polymerase complex are incubated and then mixed with prepared compounds plus UTP, which acts as a nucleotide substrate. The generated dsRNAs are then detected by addition of a fluorescent intercalant agent (Picogreen) [44,60]. Picogreen is originally developed for staining and quantifying dsDNA but can bind dsRNAs in comparison to ssRNAs. The effect of putative compounds is shown by the fluorescence readouts (Figure 3). Besides the Picogreen dsDNA fluorescent reagent, previous studies have shown other fluorescent dyes used to discriminate dsRNAs from ssRNAs. For instance, SYTO 9 is employed to monitor real-time polymerization of the Zika virus RdRp [61], while Quantiflour dsDNA system is shown to be an ideal dye for quantification of dsRNAs formed in bacteriophage Φ6 RdRp reactions [45]. Recently, a reproducible and reliable HTS model has been developed to identify SARS-CoV-2 RdRp inhibitors in a 96-well plate, which combines a uracil-rich self-priming RNA with fluorometric measurement of dsRNA. This model has demonstrated two non-nucleoside analog inhibitors (NNIs) C646 and BH3I1 block the RdRp activity with an IC_50_ value of 14.31 μM and 56.09 μM respectively and has been applied to screen for potential RdRp inhibitors from a custom synthetic chemical and natural product library, offering an efficient platform for identifying and confirming novel molecules targeting the SARS-CoV-2 RdRp, especially NNIs [46].

### Strand displacement assay

**Figure 4.**
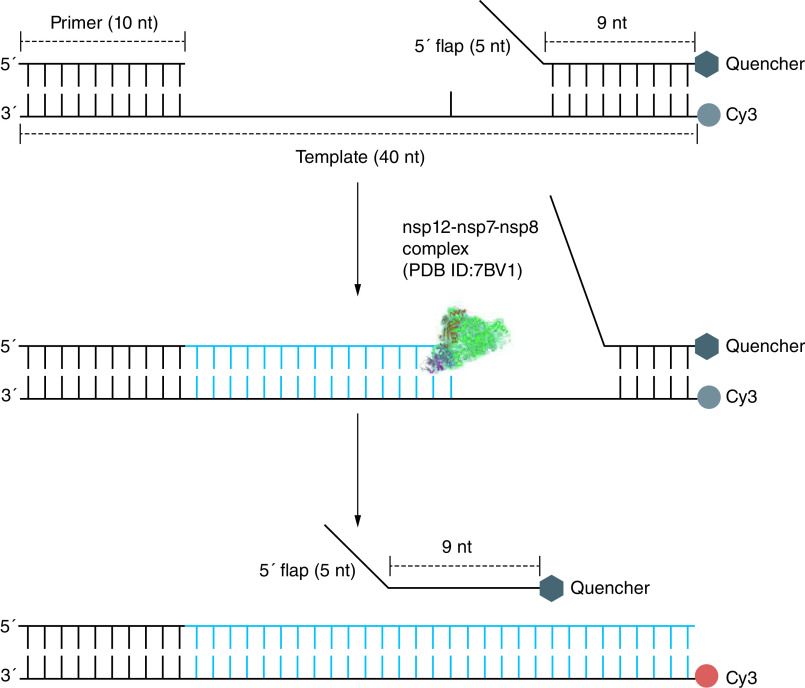
Strand displacement assay. The RNA template is annealed with a primer and a quencher strand. As activating polymerization reaction, the quencher strand is displaced by newly synthesized products under the drive of RdRp and thus unable to quench Cy3 fluorescence. RdRp: RNA-dependent RNA polymerase.

Bertolin *et al.* designed a fluorescence resonance energy transfer-based strand displacement assay amenable to HTS for discovering small molecules targeting SARS-CoV-2 RdRp in a 384-well plate [47]. The activity of strand displacement for DNA/RNA polymerases refers to the ability to displace the pre-existing DNA/RNA fragment downstream while synthesizing the new fragment [62,63]. Utilizing this activity of RdRp, the RNA substrate is formed which consists of a RNA template labeled with a Cy3 fluorophore on its 5′ end, an annealed primer, and an annealed quencher strand close to the fluorophore. After incubation of RdRp with compounds, the polymerization reactions are started by the addition of substrate mix. When RdRp extends the primer, it can drive strand displacement of the quencher strand downstream leading to growing fluorescent signals (Figure 4). The authors have identified three novel NNIs against RdRp (C646, BH3I-1 and GSK-650394) and demonstrated the anti-RdRp efficacy of suramin and suramin-like molecules [47].

On the other hand, it is noteworthy that the above cell-free assays employ nsp12 along with its cofactors, nsp7 and nsp8. The cofactors could act as a mixture by simply mixing an optimized ratio of purified nsp7 and nsp8 [41,42,64] or as a fusion protein by introducing a 6 × histidine or GSGSGS linker between the two subunits [36,44], thus supporting the constitution of the minimal RNA polymerase complex.

### Cell-based reporter assay

**Figure 5.**
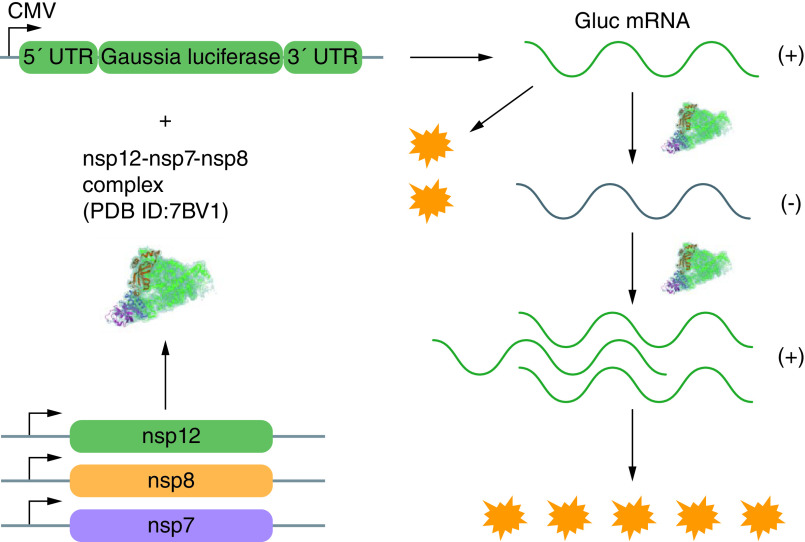
Cell-based reporter assay. The plasmid CoV-Gluc is the expression reporter vector with the *Gluc* gene flanked by 5′ and 3′ UTRs of the SARS-CoV-2. In cells transfected with CoV-Gluc, nsp12, nsp7 and nsp8 plasmids, RNA polymerase synthesizes the negative strand of the vRNA and subsequently amplifies the synthesis of Gluc mRNA and Gluc protein. Gluc: Gaussia-luciferase; PDB ID: Protein Data Bank ID; UTR: Untranslated region.

The cell-free assays have presented several insuperable obstacles. First, the assays require conversion of the NIs prodrugs into 5′-triphostrates, which is a normal metabolic process in cells [65]. However, *in vitro* synthesis of active triphosphate forms is a complicated and time-consuming chemical process, which impedes the development of the cell-free assays for drug screening [65]. Besides, it is difficult to assess cellular permeability of examined compounds without a cell system. In addition, purified recombinant enzymes do not always undergo proper folding and thus might have incorrect structural conformation [66]. To circumvent the challenges associated with the cell-free assays, a cell-based reporter assay targeting SARS-CoV-2 RdRp has been proposed on the basis of previous effective systems [67,68]. The reporter system is divided into two components: a Gaussia-luciferase (Gluc) reporter plasmid and plasmids bearing RNA synthesis genes made up of nsp7, nsp8 and nsp12 [48]. The Gluc expression reporter plasmid has the *Gluc* gene flanked by 5′ and 3′ untranslated regions of the SARS-CoV-2, which produces positive-strand of vRNA encoding the Gaussia luciferase [65]. Transcription of the *Gluc* gene is modulated by CMV promoter [65]. Within the HEK293T cells that are transfected with appropriate ratios of CoV-Gluc, nsp12, nsp7 and nsp8 plasmids, the untranslated regions of the Gluc mRNA are recognized by the SARS-CoV-2 RdRp and then the mRNA is amplified by the viral RdRp. The amplification occurs through the minus-strand stage, initiating extensive elevation of the Gluc mRNA and protein (Figure 5) [48,49]. After incubation with selected compounds, cell supernatant is collected and used to measure the Gluc activity, as the relative activity of the SARS-CoV-2 RdRp is proportional to the intensity of the luciferase activity [49]. Statistical analyses show that the Z’ factor, a frequently used parameter for validating the sensitivity and accuracy of HTS assays [69], meets the threshold required for the techniques [65]. Taken together, the cell-based assay with *Gluc* as the reporter gene is a feasible platform for testing viral polymerase inhibitors in HTS systems. This is achieved by simply detecting the luciferase signals.

## Conclusion

So far, the urgent need to contain the SARS-CoV-2 pandemic has propelled the rapid advances in drug development strategies. RdRp is deemed as the Achilles’ heel in the SARS-CoV-2 due to its essential functions in viral lifecycle. Owing to high-resolution cryo-EM structural analysis, minimal RNA synthesizing machinery has been elucidated as a complex between nsp7 with nsp8 and nsp12. Drug discovery assays targeting this tripartite complex have been developed at an unprecedented rate. Undeniably, a variety of computational screening approaches including ligand-based and structure-based drug screening have become a promising alternative for drug discovery processes to speed up the development of candidates against RdRp [70]. Several reviews have presented *in silico* studies on identifying potential RdRp inhibitors [71–73]. However, these compounds determined by *in silico* strategies still require further experimental evaluations to test their antiviral potency. For *in vitro* assays, traditional methods employ ^32^P-labeled nucleotides or primers that integrate into nascent RNA strands to analyze the enzymatic characteristics of the viral RdRp [45]. Although highly sensitive, these radioactive methods are limited because of the health concerns and environmental safety. The limitations hinder longer-term development and broader application of these methods. In contrast, the primer extension assay, fluorescence-based assay and strand displacement assay present safer alternatives since they do not utilize radioactive substances. The assays use fluorescently labeled RNA substrates and the fluorescent dye, which do not require specialized equipment and handling procedures.

Non radioactivity is a common attribute associated with the above cell-free assays and they are beneficial in diverse ways. First, as a popular biochemical method to characterize the activity of the viral polymerases, primer extension assay provides a molecular basis to better comprehend the mechanism of action of promising antiviral compounds that target the SARS-CoV-2 RdRp. For instance, Sofosbuvir inhibits the synthesis of SARS-CoV-2 RNA as a chain terminator by polymerase extension reaction [74]. In addition, Gordon *et al.* observed that incorporation of RDV-TP at position *i* causes termination of RNA synthesis at position *i*+3, which is regarded as delayed chain-termination pattern [75]. Subsequent studies confirmed similar behavior of RDV-TP, and pointed that the RDV-TP-specific delayed chain-termination pattern varies with different template sequences [41,76]. On the other hand, miniaturization and automation in the fluorescence-based assays enhance the discovery and identification of SARS-CoV-2 RdRp inhibitors from small-molecule compound libraries. It is feasible to anticipate increased output by scaling up the formats to 384 and 1536-well plates. Along with the benefits of the small volume of the reaction mixture and minimal operation, the assay utilizes economical homopolymeric RNA as a template instead of labeled synthetic heteropolymeric RNA [61]. Additionally, strand displacement assay enables real-time detection of RdRp activity avoiding the limitation of depending only on end point values [47].

In contrast to cell-free assays, a typical cell environment is the most significant advantage associated with the cell-based reporter assay. Instead of preparing the triphosphate forms of the NIs, the cell-based reporter assay is capable of directly testing nucleotide prodrugs. In addition, it is possible to simultaneously evaluate cellular permeability and cytotoxicity of the drugs. The assay also mimics a physiological environment to form the RNA synthesis complex, evading the often tedious *in vitro* recombinant protein purification protocols. To improve the consistency and convenience of the experimental design at the level of transient transfection, the next process is to establish stable cell lines that could co-express all of the integral components involved in the replication and transcription of the SARS-CoV-2 RNA genome [65].

Collectively, we summarize cell-free and cell-based assays for detecting the enzymatic activity of SARS-CoV-2 nsp12–nsp8–nsp7 complex and evaluating the inhibitory effect of prospective anti-RdRp compounds. The emergence of developed and proven techniques with the target of SARS-CoV-2 RdRp has breathed new life into the course of antiviral candidate exploring, though deeper investigation is awaited. There is no dispute that HTS assays lie at the heart of the screening from the reservoir of FDA approved drugs for treatment of other diseases, a series of novel synthesized inhibitors and the molecules which have been shown potential antiviral performance in computational analyses. Furthermore, structural and functional insights into coronavirus replication and transcription will contribute to the improvement and optimization of *in vitro* screening schemes which can be formatted into HTS conveniently.

## Future perspective

Recent studies have highlighted that the nsp14–nsp10 complex, responsible for RNA proofreading which is requisite for attainment and maintenance of large nidovirus genomes [77], should be incorporated into the screening assays that focus on the RdRp [49,65]. In complex with its activator nsp10, nsp14 has been shown to increase the fidelity of viral replication through its exoribonuclease domain, posing a relatively high resistance threshold of the RdRp to NA compounds [78,79]. By simultaneously introducing the nsp14–nsp10 and RdRp into the cell-based reporter assay, it was shown that the exoribonuclease complex, nsp14–nsp10, intensifies the resistance property of SARS-CoV-2 RdRp to NA inhibitors [65]. Therefore, considering enhanced tolerance of RdRp conferred by the proofreading capacity of exoribonuclease in seeking anti-RdRp compounds, it is necessary to expand the minimal RNA polymerase complex (nsp12–nsp8–nsp7 complex) to the nsp12–nsp8–nsp7–nsp14–nsp10 super complex.

In addition to testing the efficacy of a single candidate, these reviewed approaches could be applied to evaluate the additive or synergistic interactions between two or more potent RdRp inhibitors with different modes of action to suppress potential drug resistance and enhance antiviral potency. For instance, a combination of a NNI, corilagin (RAI-S-37) and a NI, remdesivir, exhibits additive anti-RdRp activity in the cell-based reporter assay [49]. Therefore, combined drug testing for SARS-CoV-2 RdRp might be a novel strategy for development of efficient inhibitor screening assays.

**Executive summary**

- The SARS-CoV-2 RNA-dependent RNA polymerase (RdRp) is acknowledged as a valuable target for drug development.

**The functions of SARS-CoV-2 RdRp**

- RdRp plays an indispensable role in replication and transcription of the SARS-CoV-2 genome.
- RdRp has two functional domains: a polymerase domain and a nidovirus N-terminal RdRp-associated nucleotidyltransferase (NiRAN) domain.

**The RdRp is dependent on nsp7 & nsp8 in RNA synthesis**

- Together with cofactors, nsp7 and nsp8, RdRp also referred to as nsp12 forms the basic RNA synthesizing machinery, the nsp12–nsp8–nsp7 complex.
- The cryo-electron microscopy structures give more insights into the RNA synthesizing complex.

**Assays used to screen for inhibitors of SARS-CoV-2 RdRp**

- Primer extension assay is mainly used for discovering nucleotide-based inhibitors of RdRp.
- Fluorescence-based assay features a fluorescent intercalant agent which is capable of quantifying dsRNA.
- Cell-based reporter assay characterized by a cell environment circumvents several difficulties associated with the cell-free assays.
- Strand displacement assay is a fluorescence resonance energy transfer-based strand displacement assay amenable to high-throughput screening for discovering small molecules targeting SARS-CoV-2 RdRp in a 384-well plate.

**Conclusion**

- It is necessary to develop high-throughput screening assays for evaluating the efficiency of RdRp inhibitors.

**Future perspective**

- The nsp14–nsp10 complex should be considered into the screening assays with the target of RdRp.
- It can be applied to test combined inhibitory performance utilizing two or more potent RdRp inhibitors with these verified techniques.
